# Patient‐Reported Outcome Measures and Clinical Performance in Hemophilia: Implications for Person‐Centered Care

**DOI:** 10.1111/hae.70363

**Published:** 2026-07-31

**Authors:** Tatyane Oliveira Rebouças Brito, Luiz Ivando Pires Ferreira Filho, Luany Elvira Mesquita Carvalho, Francisco Josimar Girão Júnior, Pedro Everson Alexandre de Aquino, Chiara Bezerra Barreira, Lucilane Maria Sales da Silva

**Affiliations:** ^1^ Doctor of Clinical Assistance from the State University of Ceará and nurse at the Hematology and Hemotherapy Center of Ceará Fortaleza Brazil; ^2^ Hematologist at the Hematology and Hemotherapy Center of Piauí and Professor at the Federal University of Piauí Teresina Brazil; ^3^ Hematologist at the Hematology and Hemotherapy Center of Ceará Fortaleza Brazil; ^4^ Federal University of Ceará Fortaleza Brazil; ^5^ Biomedical scientist at the Hematology and Hemotherapy Center of Ceará Fortaleza Brazil; ^6^ Physiotherapist at the Hematology and Hemotherapy Center of Ceará Fortaleza Brazil; ^7^ Professor in the Postgraduate Program in Clinical Assistance at the State University of Ceará Fortaleza Brazil

**Keywords:** Haemophilia, Patient‐Reported Outcome Measures (PROMs), person‐centered care, Joint health

## Abstract

**Objectives:**

To examine the association between patient‐reported outcome measures (PROMs) and objective measures of clinical performance in children, adolescents, and adults with haemophilia, highlighting their implications for person‐centered care.

**Background:**

Advances in haemophilia treatment have reduced bleeding rates, increasing the relevance of patient‐reported outcome measures (PROMs) to capture the real impact of the disease on a person's life. However, the integration of PROMs into clinical practice remains limited, despite the growing emphasis on person‐centered, participatory, and preference‐sensitive care.

**Methods:**

Observational, cross‐sectional, and analytical. 111 participants with haemophilia A (33 aged 8–17 years; 78 aged 18 years or older) were assessed at a Brazilian comprehensive haemophilia care centre. Patient‐reported outcome assessment instruments (PROMs) included EQ‐5D, VAS, PEDHAL, or HAL, depending on age. Clinical performance was assessed using the HJHS and FISH. Spearman correlations were applied.

**Results:**

The PROMs showed moderate to strong correlations, clinically consistent with joint health and functional independence in both age groups. In children/adolescents, the PEDHAL showed a strong correlation with the HJHS. In adults, the HAL and EQ‐5D‐3L demonstrated the highest agreement with the HJHS and FISH. These findings indicate high convergent validity and reinforce patients' subjective perception as an accurate reflection of their functional status.

**Conclusions:**

The systematic use of these instruments can contribute to longitudinal monitoring, personalization of therapeutic strategies, and improvement in the quality of care, especially in adult populations that present a higher disease burden and a poorer perception of quality of life over time.

## 1 Introduction

Haemophilia A and B are complex hereditary coagulopathies resulting from mutations in the genes that encode coagulation factors VIII and IX, respectively. With X‐linked recessive inheritance, both share severe clinical manifestations, characterized predominantly by recurrent hemarthrosis and intramuscular bleeding which, if not properly managed, culminate in disabling chronic arthropathy [1, 2].

In Brazil, the country has established itself as having the fourth largest population of patients with haemophilia in the world. Data from the Ministry of Health indicate a universe of 29,785 individuals with hereditary coagulopathies, of which 11,384 have haemophilia A and 2234 have haemophilia B [3].

The evolution of the therapeutic arsenal, and notably the greater availability of long‐lived half‐life factor concentrates, subcutaneous haemostatic agents, and the implementation of personalized prophylaxis protocols, has allowed high‐income countries to achieve a life expectancy comparable to that of the general population, with a significant reduction in joint morbidity [4, 5].

Despite the reduction in annual bleeding rates (ABR), this isolated indicator has become insufficient to measure the quality of care and the value delivered to the patient. Currently, the bleeding rate is seen as an outcome that must be complemented by measures that capture the interference of pain, functionality and health‐related quality of life (HRQoL) [6]. The integration between objective clinical methodologies and patient‐reported outcomes (PROMs) therefore constitutes the central axis of person‐centered care [7].

This study was guided by the following question: although the importance of PROMs is recognized, what is the evidence gap regarding the correlation between these PROMs and clinical measures in different age groups in the Brazilian population?

The aim of this study was to evaluate the relationship between patient‐reported outcome (PROMs) measures and clinical, joint, and functional performance in different age groups of people with haemophilia A and B followed at a reference centre in northeastern Brazil.

### 1.1 Methodology

This observational, cross‐sectional, and analytical study was conducted in accordance with the *STROBE guideline* (Strengthening the Reporting of Observational Studies in Epidemiology), ensuring methodological rigor in reporting epidemiological research [8]. 111 participants were included, comprising 33 children and adolescents (8 to 17 years) and 78 adults (≥18 years). All individuals had a laboratory‐confirmed diagnosis of haemophilia and were under regular follow‐up at the service where the study was conducted. Data collection took place during routine elective consultations, in which participants answered questionnaires after clinical evaluation by healthcare professionals.

Clinical assessments and self‐reported questionnaires were conducted at the same meeting; however, the physiotherapists responsible for the clinical assessment did not have access to the PROM responses during data collection. Similarly, participants did not receive feedback or comments regarding the HJHS or FISH before completing the questionnaires, minimizing potential influence between the measures.

Informed Consent was duly applied to all participants or their legal guardians. The study was approved by the Research Ethics Committee, under opinion number 70723823.5.0000.8152.

### 1.2 Instruments and Measurements Used

Patient‐reported outcome measures (PROMs) were selected according to the participants' age range, using validated instruments widely used in the functional and quality of life assessment in haemophilia.

### 1.3 For Children and Teenagers (8–17 years old)

EQ‐5D‐Y: Generic quality of life instrument adapted for the pediatric population; VAS (Visual Analogue Scale): A visual analog scale from 0 to 100, which represents the subjective perception of health status; PEDHAL (Pediatrics) Haemophilia Activity List (HAL): Pediatric version of the HAL, expressed as a normalized percentage, reflecting the functional performance perceived by the patient.

For adults (18 years and older)EQ‐5D‐3L: Quality of life questionnaire composed of five dimensions and three severity levels per item; VAS (Visual Analogue Scale): Visual analog scale of general health perception; HAL (Haemophilia Activity List): specific instrument for haemophilia, which assesses functionality in daily activities as a normalized percentage. In all instruments, higher values correspond to better quality of life and greater functionality.

#### 1.3.1 Clinical Performance Measures

Objective clinical outcomes were assessed by physiotherapists at the service. The data were recorded in a standardized database.

The HJHS (Haemophilia Joint Health Score, version 2.1): A clinical score that assesses joint health in six joints (knees, ankles and elbows), ranging From 0 (normal joint) to 124 (severe damage); FISH (Functional Independence Score in Haemophilia): A functional measure that assesses independence in seven daily activities, With a total score Between 7 and 32, where higher values indicate greater functional independence

### 1.4 Statistical Analysis

Statistical analyses were performed using Python software (version 3.11), using the *pandas, numpy* and *scipy.stats libraries* [9]. Initially, descriptive analysis of quantitative variables was performed, including mean, standard deviation, median, minimum and maximum values [10]. The normality of the data distribution was assessed by graphical inspection and by the Shapiro–Wilk test [11], showing an absence of normality.

Therefore, non‐parametric tests were employed. The association between self‐report measures (PROMs) and clinical performance measures (HJHS and FISH) was analysed using Spearman's correlation coefficient [12]. The results were presented as correlation coefficients (*ρ*) and respective *p*‐values, adopting *p* < 0.05 as the level of statistical significance. The magnitude of the correlations was interpreted according to previously established criteria according to Cohen: weak (0.10–0.29), moderate (0.30–0.49) and strong (≥0.50) [13].

## 2 Results

33 patients were included in the study pediátricos (8–17 anos) e 78 adultos (≥18 anos), of whom 92 (82.9%) had severe haemophilia A and 19 (17.1%) had moderate haemophilia A, all male. In the adult group, 70 participants (89.7%) were on prophylaxis, while among children and adolescents, all (100%) were under prophylactic treatment and 8 (7.2%) patients had a positive inhibitor against factor FVIII.

The data presented highlight striking differences between age groups, reflecting the clinical evolution of haemophilia throughout life. Children and adolescents (8–17 years) showed better outcomes in all instruments evaluated, with low scores on the HJHS (mean of 3.7), high levels of functionality (FISH: 31.7) and quality of life close to ideal (EQ‐5D‐Y: 0.96), in addition to a very positive perception of health (VAS: 95).

In contrast, adults (≥18 years) demonstrated substantial impairment, with higher scores for joint damage (HJHS: 32.8), reduced functional capacity (FISH: 25.7; HAL: 72.5), and worse perceived quality of life (EQ‐5D‐3L: 0.49; VAS: 79). These findings suggest a progressive accumulation of joint damage and functional impact over time, possibly associated with the historically lower availability of prophylactic strategies in older individuals. Additionally, the greater variability observed in the adult group indicates relevant clinical heterogeneity. In Table 1, we observe the characterization and objective clinical measures of the participants.

**TABLE 1:** Characterization and objective clinical measurements of the participants. Fortaleza, Brazil. 2025.

Variable	Group	*n*	Average	DP	Median	Mín.–Máx.
8–17	Age (years)	33	12,5	2.4	12,5	8–17
	VAS (0–100)	33	95,0	5.2	95	80–100
	Total HJHS	33	3.7	5.3	2	0–20
	Standardized PEDHAL	33	97,4	4,7	99,6	75,8–100
	FISH	33	31,7	1.0	32	28–32
	EQ‐5D‐Y	33	0,96	0,08	1,00	0,787–1,00
≥18	Age (years)	78	34,9	11.0	33,5	18–66
	VAS (0–100)	78	79,0	15.1	80	10–100
	Total HJHS	78	32,8	16.1	29	6–74
	Standardized HAL	78	72,5	21.0	73,8	15,6–100
	FISH	78	25,7	5.2	26	12–32
	EQ‐5D‐3L	78	0,49	0,32	0,52	−0,70–1,00

Abbreviations: EQ‐5D‐3L, EuroQol 5 Dimensions; FISH, Haemophilia Functional Independence Index; HAL, Haemophilia Activity List; HJHS, Haemophilia Joint Health Index; SD, Standard Deviation; VAS, Visual Analogue Scale.

In Table 2, we observe the correlation between the reported measures and the clinical performance of the pediatric group.

**TABLE 2:** Correlation Between PROMs and clinical performance. Group aged 8 to 17 years. Fortaleza, Brazil

Correlated variables	n	*ρ* (Spearman)	valor *p*	Interpretation
VAS × Total HJHS	33	−0,552	0,0009	Better perception → less joint damage
PEDHAL × HJHS Total	33	−0,656	0,00003	Greater function → less impairment
VAS × PEDHAL	33	0,567	0,0006	Perception and function go hand in hand.
EQ‐5D‐Y × HJHS Total	33	−0,070	0,700	Not significant
EQ‐5D‐Y × FISH	33	0,313	0,076	Positive trend

Abbreviations: EQ‐5D‐Y, EuroQol 5 Dimensions – Youth; FISH, Haemophilia Functional Independence Index; HJHS, Haemophilia Joint Health Index; PEDHAL, Pediatric List of activities related to haemophilia; VAS, Visual Analogue Scale.

Spearman's correlation analysis revealed statistically significant and clinically relevant associations between perceived health, functionality, and joint damage. A moderate negative correlation was observed between VAS and total HJHS (*ρ* = −0.552; *p* = 0.0009), indicating that better perceived health is associated with less joint impairment. Similarly, the correlation between PEDHAL and total HJHS was negative and of greater magnitude (*ρ* = −0.656; *p* = 0.00003), suggesting that greater functional capacity is strongly related to less joint damage. Additionally, a moderate positive correlation was identified between VAS and PEDHAL (*ρ* = 0.567; *p* = 0.0006), demonstrating that subjective perceived health follows objective functionality.

On the other hand, there was no significant correlation between the EQ‐5D‐Y and the total HJHS (*ρ* = −0.070; *p* = 0.700), and the association with FISH showed only a positive trend, without statistical significance (*ρ* = 0.313; *p* = 0.076). These findings suggest that, in this age group, haemophilia‐specific instruments, such as the HJHS and PEDHAL, are more sensitive in capturing relevant clinical changes, while generic quality of life measures, such as the EQ‐5D‐Y, may have lower sensitivity, configuring a ‘ceiling effect’ of the disease. This interpretation is supported by the observation that 75.8% of participants achieved the maximum score on the EQ‐5D‐Y, exceeding the 15% limit proposed in the literature for characterizing this phenomenon. This suggests a limited capacity of the EQ‐5D‐Y to discriminate subtle differences in health and functionality in children and adolescents with haemophilia who have good clinical control [14].

Table 3, for the group aged ≥18 years, shows the correlations between PROMS and clinical performance, which were also statistically significant and consistent with the global literature.

**TABLE 3:** Correlation between PROMS and clinical performance—Group ≥18 years. Fortaleza‐CE. Brazil.

Correlated variables	n	*ρ* (Spearman)	valor *p*	Interpretation
VAS × Total HJHS	78	−0,323	0,004	Significant moderate negative correlation
HAL × HJHS Total	78	−0,487	< 0,0001	Greater function → less joint damage
EQ‐5D‐3L × HJHS Total	78	−0,422	0,0001	Better quality of life → lower HJHS
VAS × FISH	78	0,370	0,0008	Better perception → better function
HAL × FISH	78	0,593	1,1 × 10^−^^8^	Strong positive correlation
EQ‐5D‐3L × FISH	78	0,639	3,1×10^−^^10^	Strong and highly significant positive correlation

Spearman's rank correlation coefficient. *p* = statistical significance value (= 0.05).

Abbreviations: EQ‐5D‐3L, EuroQol 5 Dimensions; FISH, Haemophilia Functional Independence Index; HAL, Haemophilia Activity List; HJHS, Haemophilia Joint Health Index; VAS, Visual Analogue Scale.

Quality of life (EQ‐5D‐3L), function (HAL), and general health perception (VAS) scores showed negative correlations with the total HJHS, ranging from moderate to strong (*ρ* between −0.32 and −0.49; *p* < 0.01). These results indicate that greater joint damage is associated with worse perceived quality of life and physical function. On the other hand, correlations with FISH were positive and of strong magnitude, especially for HAL (*ρ* = 0.593; *p* < 0.001) and EQ‐5D‐3L (*ρ* = 0.639; *p* < 0.001), demonstrating that better perceived function and quality of life reflect better functional clinical performance.

## 3 Discussions

Patient‐reported outcome measures have been used to assess quality of life and health status preferences from the patient's perspective. These assessments have been the focus of haemophilia researchers, aiming to establish international standards and facilitate comparisons between studies. On the other hand, clinical measures alone cannot adequately characterize the impact of the disease on patients. Patient preferences are numerical measures that represent the value assigned to a specific health status [15]. This intersection characterizes person‐centered care.

This growing emphasis on patient‐centered outcomes in haemophilia has been driven by international initiatives such as the International Consortium for Health Outcomes Measurement (ICHOM). The study conducted by van Balen proposed a standardized set of outcomes that integrates clinical measures and patient‐reported outcomes, including pain, physical function, social participation, and mental health. This approach reinforces the need to assess haemophilia comprehensively and longitudinally, considering the impact of the disease on daily life [16].

Joint health is one of the main causes of low quality of life in adults with haemophilia. As haemophilia causes bleeding in the joints, it leads to progressive destruction of the joint structure, ultimately resulting in chronic pain, joint dysfunction, and disability. Corroborating our study, quality of life, pain, and physical function are the most evaluated domains [17], while psychosocial and mental health aspects remain underexplored, showing a need for greater standardization and validation of these metrics in different cultures to improve and refine patient‐centered care.

Correlations between patient‐reported outcome measures (PROMs) and clinical performance indicators confirmed the consistency and applicability of the instruments used. In both groups, the expected direction of the associations was observed: PROMS ↑ ↔ FISH ↑ (better perception associated with better function) and PROMS ↑ ↔ HJHS ↓ (better perception associated with less joint damage). The PEDHAL stood out in pediatrics as the most sensitive instrument, while in the adult group the HAL and EQ‐5D‐3L scales showed the strongest correlations, reinforcing their convergent validity. A longitudinal analysis, and despite therapeutic advances, patients with haemophilia, especially those over 50 years of age, continue to report worse quality of life and physical function compared to the general population, according to Johnson's study, corroborating the results of this study [6].

On the other hand, there is a disability paradox manifesting itself when individuals with physical limitations report high levels of HRQoL, resulting in a discrepancy between personal perception of health and objective clinical status. This phenomenon is influenced by the patient's adaptation to the disability and the use of coping strategies, which can lead to an underestimation of the real burden of the disease as observed in those under 18 years of age and also observed in Burkner study [18].

Other studies, P‐FiQ and B‐HERO‐S, which used PROMS instruments to assess pain, functional limitations, anxiety, depression and difficulties in accessing treatment, showed that even less severe forms of the disease have a significant impact on patients' lives, reinforcing the need for person‐centered approaches [18, 19]

Adherence to treatment and its effects on clinical and humanistic outcomes, including PROMs such as pain, HRQoL, work/school productivity and cognitive function, suggests that greater adherence to treatments is associated with better patient‐reported outcomes, reinforcing the importance of personalized follow‐up and health education strategies [20].

The ICHOM (International Consortium for Health Outcomes Measurement) created and validated a list of sets of clinical outcome indicators that focus on two domains: musculoskeletal health and function and safety; and patient‐reported outcomes identified five domains: Bleeding frequency, pain, mobility and physical activities, health‐related quality of life, and satisfaction. Implementing this value‐based approach would allow for a more robust assessment of the quality of care in haemophilia, within a framework of continuous treatment improvements, with potential added value for patients [21].

Personalized treatment strategies, which align treatment with each patient's individual goals, initiated at the time of diagnosis and maintained throughout life, are important for optimal outcomes. These strategies are facilitated by a multidisciplinary team and supported by patient education.

The incorporation of patient‐reported outcomes (PROMs) into clinical practice in haemophilia can be structured systematically and aligned with the principles of person‐centered care. A viable approach involves implementing an integrated workflow with the application of validated instruments at key moments in care (routine multidisciplinary consultations, initiation or change of therapy), integrating these metrics into clinical systems, such as physical or electronic medical records that allow for this longitudinal visualization of scores; and transforming the data into information that assists in decision‐making, with discussion of the results between the multidisciplinary team and the patient, guiding individualized therapeutic adjustments.

This model promotes continuous monitoring, early identification of clinical deterioration, and can generate greater patient engagement in care, as it contributes to better communication between professional and patient, greater sensitivity in detecting clinical changes, and support for decision‐making. Thus, the structured incorporation of PROMs into clinical practice represents a strategic advance to improve the quality of care in haemophilia, especially in adult populations with a higher disease burden.

### 3.1 Study Limitations

Because this was a cross‐sectional observational study conducted at a single referral centre, a convenience sample was used, composed of all eligible patients seen during the data collection period. Post‐hoc statistical power analysis indicated that approximately 47 participants would be needed to detect moderate correlations (*ρ* = 0.40), with *α* = 0.05 and a power of 80%. Therefore, the total sample of 111 participants was considered adequate for the main analyses, although the pediatric subgroup (*n* = 33) has less power to identify associations of small magnitude. Limitations include the cross‐sectional design, which does not allow for causal inferences, and the fact that the study was conducted at a single centre.

Variables such as haemophilia severity, presence of inhibitors, and prophylactic therapeutic regimen can influence both objective clinical outcomes and patients' reported perception of health, functionality, and quality of life. Although the high proportion of participants on prophylaxis (89.7% of adults and 100% of children/adolescents) reflects contemporary clinical practice and may have contributed to the good results observed, especially in the pediatric group, it was not possible to assess the independent impact of these variables due to the cross‐sectional design and the relatively homogeneous distribution of the sample. Previous studies demonstrate that disease severity, the development of inhibitors, and access to prophylactic strategies are associated with significant differences in joint health, functionality, and quality of life, and may modify the magnitude of the associations between PROMs and clinical measures. Therefore, future investigations with larger samples and adjusted analytical models should explore the role of these factors in the relationship between patient‐reported outcomes and clinical performance indicators.

## 4 Conclusions

The results of this study highlight significant differences between children/adolescents and adults with haemophilia, particularly regarding the perception of quality of life, functionality, and joint impairment. While younger individuals presented better scores on all evaluated instruments, adults demonstrated a higher disease burden, with a worse perception of health and greater functional limitation. These findings reflect the progressive nature of haemophilia, in which recurrent bleeding episodes throughout life lead to the development of chronic arthropathy, directly impacting functional capacity and well‐being.

In this context, PROMs have become essential tools in the assessment of haemophilia, as they allow the measurement of dimensions that are not fully captured by traditional clinical indicators. In the present study, the strong correlation between functionality, joint damage, and health perception reinforces the relevance of the combined use of these tools for a more patient‐centered assessment.

These results should also consider the advancement of therapeutic strategies over the last few decades. The widespread adoption of prophylaxis, especially in children and adolescents, has contributed to the reduction of bleeding and prevention of joint damage, which is reflected in the better outcomes observed in this group. On the other hand, many adults were exposed, at early stages of life, to less effective therapeutic regimens, which may explain the greater functional impairment and poorer quality of life observed.

Finally, these findings reinforce the importance of incorporating person‐centered care models, in which the patient's perspective is fundamental to the evaluation of health outcomes. This reinforces international initiatives, such as the ICHOM proposal, emphasizing the need to integrate PROMs (Prognosis, Results, and Medications) with traditional clinical indicators, promoting a more comprehensive approach. The set of outcomes developed for haemophilia highlights domains such as pain, physical function, mental health, and social participation, which are still gaps and unmet needs in clinical practice. Therefore, the systematic use of these instruments can contribute to longitudinal monitoring, the personalization of therapeutic strategies, and the improvement of the quality of care, especially in adult populations that present a higher disease burden and a poorer perception of quality of life over time.

## Funding

The study received financial support from the Coordination for the Improvement of Higher Education Personnel – Brazil (CAPES) and and technical support from the Pró‐Hemo Saude Institute ‐ IPH.

## Ethics Statement

This study was conducted in accordance with the principles of the Declaration of Helsinki and followed the STROBE recommendations for observational studies. Ethical approval was obtained from the Research Ethics Committee under approval number 70723823.5.0000.8152.

## Consent

This study was carried out following the principles of the Declaration of Helsinki. The research protocol got the green light from the Research Ethics Committee at the Centro de Hematologia e Hemoterapia do Ceará (HEMOCE) (CAAE 70723823.5.0000.8152). Everyone who took part gave their written consent beforehand. Confidentiality and anonymity were kept throughout the study.

## Conflicts of Interest

The authors declare no conflicts of interest.

## Data Availability

The data supporting the conclusions of this study are available in the supplementary material to this article. The generated and analysed datasets were previously anonymized, ensuring the confidentiality and privacy of the participants, and their use was approved by the Research Ethics Committee.
